# Angina Pectoris

**Published:** 1905-05-20

**Authors:** 


### ANGINA PECTORIS.

An excellent and comprehensive account of this symptom is offered by a recent lecture of Dr. Graham Steell.[1] The disease or diseases of which angina pectoris is the expression are so notoriously insidious in their manifestations, and the pains expressive of them so similar at times to the comparatively trifling "rheumatic" aches of later life, that the author does well to dwell at some length on the character and explanation of the "referred pains" of visceral disease, to a proper appreciation and interpretation of which Dr. Head has so largely contributed. The accepted explanation of these pains is thus briefly enunciated by Dr. Steell. "Impressions arising in the heart, having arrived at the associated segment of the cord, undergo diffusion in the grey matter and seem to the patient to proceed from the fibres of the somatic spinal system with which the segment in question is directly connected, so that in the cerebrum, where alone pain can be felt by the Ego, the pain of angina pectoris is referred to the parts corresponding with the afferent supply of the grey matter into which diffusion has taken place." Now, according to Head, the ascending aorta refers its pains to the somatic distribution of the third and fourth cervical nerves with that of the first, second, and third dorsal nerves, while the ventricle and auricle are associated with the dorsal spinal segments from the second to the eighth. It is thus possible to explain the frequency with which the pains of angina pectoris radiate down the inner side of the arm and forearm, for this is the area of the somatic distribution of the first and second dorsal spinal segments.

As to the symptoms, Dr. Steell holds that pain is the essence of angina pectoris, and that without it true angina cannot be said to exist; the other essential element is a sense of impending dissolution. The distribution of the pain is variable: it commonly starts from the region of the sternum and spreads to the shoulder and thence down the inside of the arm to the ulnar side of the hand. But occasionally it is peripheral to start with, and spreads centripetally: in these cases the danger of misinterpretation is great, for the patient will complain of "rheumatism." A case in point from our own experience may be given in passing. A gentleman of 60 complained that he had for some months been subject to occasional attacks of rheumatism in both arms. On further questioning it appeared that the "rheumatism" was prone to set in just about the point of insertion of the deltoid muscles, and would appear most often during the second five minutes of active exercise. Thus he would begin a walk in perfect comfort, but after walking for two or three minutes he would become sensible of the pain, which, if he continued his exercise, would gradually become intensified until continuance in walking was impossible. But after resting for a few minutes the pain would abate and leave him capable of completing a long walk without any further symptoms of uneasiness. For two years these attacks became gradually more frequent, and the area of pain slowly spread until it was customary for each attack to involve the thorax towards its conclusion, although the deltoids remained the site of origin for each attack. As the severity of the bouts increased, the second element of angina pectoris—the sense of impending dissolution—asserted itself, and during the last few months of life there remained no question of the identity of the disease.

The curious condition above detailed was first observed in horses, and the term "intermittent claudication," which was applied to it by its first observer, a veterinary surgeon named Bouley, was appropriated for human medicine by Charcot in 1858. The symptoms in the horse, as given by Bouley, are remarkably similar to those observed in men, and since the matter has an intimate bearing upon the ætiology of angina pectoris it may not be out of place to reproduce them briefly. "The animal which has previously worked well, and may otherwise show every sign of good health, is seized while at work with lameness in one or more limbs, in general both hind-legs simultaneously. If he is given rest, this generally suffices to remove the symptoms. At the beginning of a renewed attempt, however, the lameness reappears. If the horse be beaten he will soon be seen to evince signs of great anxiety. He is seized with a general tremor and paws the ground violently; his eyes are fixed and haggard and his expression shows profound pain. Finally, he throws himself to the ground, where he may roll and throw himself about like a horse afflicted with violent colic. . . . Within 20 or 30 minutes all the symptoms subside, the attitude becomes easier, the pulse slows to normal, and the expression of pain disappears."

In the first recorded case by Bouley the lesion productive of the intermittent claudication was an obliteration of the femoral arteries, and if, as some people hold, angina pectoris is intermittent claudication of the heart, the cause of it is to be found in the almost constant presence of coronary sclerosis in sufferers from this angina. That is to say that symptoms similar to this intermittent claudication may be looked for wherever a muscle is called upon to exert itself while in a state of arterial ischæmia.

As to the cause of sudden death associated with angina pectoris, Dr. Steell suggests that it may depend upon a sudden disorganisation of Kronecker's cardiac co-ordination centre. This centre is situated at the lower limit of the upper third of the ventricular septum, and Osler has seen Kronecker puncture this spot in the dog, with the result that the heart "becomes paralysed in a state of fibrillary tremor from which it does not recover."

Although coronary sclerosis is present in a large proportion of cases of angina pectoris, Dr. Steell does not believe that the symptom has any constant anatomy, for he has seen typical angina pectoris in people who after death showed no visible lesion of the coronary arteries. None the less, for practical purposes angina pectoris, we fancy, may be regarded as an expression of coronary obstruction. This being so, it is not surprising that physical signs of cardiac disease should often be lacking where the symptoms of angina pectoris are well marked, and in this connection the author endorses the observation of Dr. Balfour that "the less there seems to be the matter with the heart the more grave is the prognosis if the attacks be at all serious."

[1] Med. Chron., March and April, 1905.

